# Assessing the Prevalence of Diabetes Distress and Determining Its Psychosocial Predictors Among Saudi Adults With Type 2 Diabetes: A Cross-Sectional Study

**DOI:** 10.3389/fpsyg.2021.759454

**Published:** 2021-12-22

**Authors:** Mohammed A. Batais, Abdulaziz F. Alfraiji, Abdulrahman Abdullah Alyahya, Ouf Abdullatif Aloofi, Mohammad Khalid Almashouq, Khalid Saeed Alshehri, Abdulrahman Mohammed Almizel, Mohammed Taraheeb Alotaibi, Fahad D. Alosaimi

**Affiliations:** ^1^Department of Family and Community Medicine, College of Medicine, King Saud University, Riyadh, Saudi Arabia; ^2^College of Medicine, King Saud University, Riyadh, Saudi Arabia; ^3^Psychiatry and Psychosomatic Medicine, Department of Psychiatry, College of Medicine, King Saud University, Riyadh, Saudi Arabia

**Keywords:** prevalence, diabetes distress, psychosocial predictors, type 2 diabetes, Saudi adults

## Abstract

In recent years, the global burden of diabetes distress has increased significantly worldwide, imposing mental health issues on patients and the healthcare system. Hence, this study aimed to estimate the prevalence of diabetes distress and determine its psychosocial predictors among Saudi adults with type 2 diabetes mellitus (T2DM). This cross-sectional, observational study was conducted at Diabetes Clinics, Tertiary Care Academic Medical Center, King Saud University Medical City, Riyadh, Saudi Arabia. The survey questionnaire was distributed to 423 participants. The sample size was 384, where the prevalence of diabetes distress was 48.5%. Based on 5% precision and a confidence interval of 95%, the response rate was 78.25% (334 respondents), among which 61.4% of respondents were females, the remaining 38.6% were males, and the mean age was 56.39 years. The mean scores for the Saudi Arabian Diabetes Distress Scale-17 (SADDS-17) subdomains including emotional burden, physician-related distress, regimen-related distress, and interpersonal distress were 2.63 ± 1.29, 2.31 ± 1.44, 2.48 ± 1.16, and 2.23 ± 1.24, respectively. Based on the World Health Organization Quality of Life Assessment Instrument, Short Version (WHOQOL-BREF) transformed scores, the quality of life was recorded as 62.7%. There was a statistically significant positive correlation between the Hospital Anxiety and Depression Scale (HADS) score (*r* = 0.287, *p* < 0.01) and the total SADDS-17 scores. The Patient Health Questionaire (PHQ) 15 scores showed significant positive correlations with the total SADDS-17 scores (*r* = 0.288, *p* < 0.01) and each of the four subdomains. Our present study revealed that diabetes distress prevalence is alarmingly high among patients in Saudi Arabia, Riyadh. Our findings provide evidence that physical symptoms, quality of life, depression, and anxiety are the notable predictors of diabetes distress.

## Introduction

Diabetes mellitus (DM) is a severe threat and a most critical public health challenge; based on the International Diabetes Federation (IDF) statistics, it is affecting 463 million people in 2019 worldwide, and this number will be increased astoundingly to 700 million (10.9%) by 2045 (IDF Diabetes Atlas, 2019). The estimated prevalence of DM in the Middle East and North Africa is 55 million in 2019 (IDF Diabetes Atlas, 2019). In particular, the prevalence of DM ranged from 25.4 to 31.6% among Saudi adults and there has been a staggering emergence of both type 1 and 2 diabetes (Al-Daghri et al., 2011; Alqurashi et al., 2011; Al-Rubeaan et al., 2015).

Diabetes is a heterogeneous disorder compounded by the breakdown of multiple systems with a high risk for cerebrovascular disease, coronary artery disease, renal failure, and foot amputation (Papatheodorou et al., 2018). Prevention and treatment of diabetes and its complications is one of the crucial factors, which worsens patients’ mental health and develops depressive and anxiety symptoms (Bădescu et al., 2016; Naicker et al., 2017). Anxiety and depression are more prevalent among patients with type 2 diabetes mellitus (T2DM) than in the general population (Tran et al., 2021). It is estimated that one in every four T2DM patients has faced depression (Khaledi et al., 2019). Regardless of their depression symptoms, persons with type 2 diabetes benefit from self-management support intervention. A text messaging strategy reduced depression symptoms in patients with an A1c of less than 8.5 percent at baseline (Mayberry et al., 2021). The links between defense mechanisms, depression, and health-related quality of life point to the possibility of personification and protagonization, which may arise over time as diabetes symptoms become more intrusive. The favorable relationship between defensive techniques and well-being measures needs to be viewed with caution (Martino et al., 2020). In addition, a counseling intervention that was successful in achieving a long-term shift in physical activity and sedentary behavior increased psychological well-being and quality of life considerably (Balducci et al., 2019); even though patients with diabetes who had symptoms of depression, but which was not clinical depression, experienced an emotional burden related to diabetes distress (Fisher et al., 2007). Compared with major depressive disorder (MDD) and depressive symptoms in diabetics, the estimated prevalence of diabetes distress was a higher percentage in the US (18%) (Fisher et al., 2008, 2010), China (42.15%) (Zhou et al., 2017), and Malaysia (49.2%) (Chew et al., 2016).

Diabetes distress is a hidden negative emotional condition caused by worries, fears, and frustrations in patients with diabetes, which is mainly associated with poorer management (Ali et al., 2006; Fisher et al., 2007, 2008, 2010). Diabetes distress is more persistent than anxiety and affective disorders (Ali et al., 2006; Fisher et al., 2007, 2008, 2010) and had a cyclical relationship between depressive symptoms and diabetes distress (Ali et al., 2006; Fisher et al., 2007, 2008, 2010). Insulin treatment had a higher prevalence of diabetes distress than oral hypoglycemic agents or diet modification (Delahanty et al., 2007). Diabetes distress is better accounted for by non-clinical factors, including coping styles and social support rather than the clinical indicators (Karlsen et al., 2012). However, a positive correlation between the hemoglobin A1c (HbA1c) and diabetes distress has been reported by several studies. Female patients who have had MDD, or experienced more negative events are at high risk of becoming distressed over time (Fisher et al., 2007, 2008, 2010).

In recent years, the global burden of diabetes distress has increased significantly worldwide, imposing mental health issues on patients and the healthcare system. In particular, high rates of T2DM are observed among the Saudi population. Unfortunately, there is little to no information related to diabetes distress in the Saudi population. This notion could open new research in psychosocial predictors associated with diabetes distress in Saudi Arabia. Hence, this study aimed to estimate the prevalence of diabetes distress and determine its psychosocial predictors among Saudi adults with T2DM.

## Materials and Methods

### Design and Participants

This cross-sectional, observational study was conducted at Diabetes Clinics, Tertiary Care Academic Medical Center (concealed text), Saudi Arabia. It is a university-affiliated government hospital that delivers free DM healthcare services, and patients benefited from rural areas and all over the country. Participants were selected using convenience sampling of adults with T2DM (age ≥ 18 years) between December 2016 and April 2017, and who could fluently read and understand Arabic. The exclusion criteria included patients who were suffering from type 1 diabetes, severe comorbid medical illnesses, severe diabetes complications, or psychiatric diseases (psychosis or dementia), and pregnant or lactating females. The participants were interviewed by trained interviewers after getting their written informed consent. Ethical approval was obtained from the research ethics committee at King Saud University.

### Sample Size

The sample size was 384, where the prevalence of diabetes distress was 48.5%. Based on 5% precision and a confidence interval of 95%, we calculated the sample size using the single proportion sample size formulae [*n* = Z2αP(1-P)/d2]. The survey questionnaire was distributed to 423 participants, with a non-response rate of 10%. The response rate was 78.25% (334 respondents); among these, 61.4% of respondents were females, the remaining 38.6% were males, and the mean age was 56.39 years.

### Measurements

A committee composed of two psychiatrists, an epidemiologist, and two family medicine physicians reviewed each component of the study’s questionnaire to ensure the relevance and applicability of each question and tested on 30 participants before the final application.

The study questionnaire distributed to participants included a socio-demographic section that contained the medical record number, which was used to acquire clinical, biochemical, medication data, and past medical history. The next part of the questionnaire included the following scales:

***The Diabetes Distress Scale (DDS-17)*** consists of 17 items divided into four domains that describe possible diabetes-related problems such as emotional burden (EB), physician-related distress (PD), regimen-related distress (RD), and interpersonal distress (ID). Based on the items scoring from 1 to 6, the scale was depicted as no distress to severe distress. The scores represent distress experienced over the last month. Scores were averaged by dividing the summed scores of each participant’s responses by the number of items in the scale. Based on their average scores, participants were categorized into two diabetes distress groups (low [<3], high [≥3]) (Polonsky et al., 2005).

***The Saudi Arabian Diabetes Distress Scale (SADDS-17)*** was first validated and translated into the Arabic language (Batais et al., 2021). Two bilingual native Arabic speakers translated this scale after gaining Lawrence Fisher’s permission (Fisher et al., 2012). One speaker had medical expertise and another speaker knew the goal of the study. After this, two researchers back-translated the Arabic into an English version in order to assess the validity of each item. A committee of two psychiatrists, an epidemiologist, and two-family medicine physicians were then formed to finalize the Arabic version of DDS-17 by adapting it to the Saudi culture.

***The Hospital Anxiety and Depression Scale (HADS)*** tool consists of 14 items, which are divided into two halves that evaluated anxiety and depression levels among participants. Each question had a range of four answers that described the severity of the exact point. A total score of 0–7 was considered within the normal range, a score of 8–10 was regarded as a borderline abnormal range, while a score within 11–21 was considered in the abnormal range. Permission to use a previously validated Arabic version of this scale was obtained (Malasi et al., 1991).

***The Visual Analogue Scale (VAS)*** is a visual self-evaluation tool that was used to evaluate participants’ compliance with diabetes management (Finitsis et al., 2016). The participants viewed a horizontal line containing small vertical lines numbered sequentially from 0 to 10, representing a range from non-compliant to fully compliant. Based on the earlier studies (Zeller et al., 2008; Alosaimi et al., 2016, 2017), participants who had scores of great than or equal to 8 were considered an adherent group and used medicine as per doctor’s instructions; scores less than 8 represent the non-adherent group.

***The World Health Organization Quality of Life Assessment Instrument, Short Version (WHOQOL-BREF)*** scale contains 26 items that evaluate the participants’ quality of life in four domains such as physical health, psychological health, social relationships, and environment during the past 2 weeks of their lives (WHOQOL, 2021). WHOQOL-BREF is a subset from the WHOQOL 100. However, it has no representative intervals. Scores in this scale are transformed into a value between 0 and 100, reflecting the lowest and highest possible scores, respectively. Prior permission to use the validated Arabic version of WHOQOL-BREF was obtained (Ohaeri and Awadalla, 2009).

***Patient Health Questionnaire (PHQ-15)*** is a scale that contains 15 items evaluating somatic symptoms perceived by patients. Answers to questions include 0, not bothered at all; 1, bothered a little; and 2, bothered a lot. The total score is calculated and ranges from 0 to 30, reflecting the severity of symptoms as minimal, low, medium, or high level (Questionnaire). Permission was obtained to use the validated Arabic version (AlHadi et al., 2017).

***The Summary of Diabetes Self- Care Activities (SDSCA)*** is a scale that contains eight items that measure the frequency of specific diabetes self-management activities in the past week. The categories included were diet, exercise, blood sugar testing, and foot care. Permission was obtained to use the validated Arabic version (AlJohani et al., 2016).

### Statistical Analysis

Descriptive statistics for continuous variables were reported as mean ± standard deviation (SD), while categorical variables were reported as frequencies. Statistical comparisons between different groups were made using independent samples *t*-test, one-way ANOVA for diabetes distress score, and chi-square test for the level of diabetes distress. Bivariate correlations were performed to find the associations between diabetes distress scores and the socio-demographic, clinical, and biochemical data. To identify independent variables, multivariate logistic regression analysis was conducted on the significant variables from the bivariate analysis. All the tests were two-tailed, and statistical significance was set at *p* < 0.05 or *p* < 0.01. All analysis was performed in SPSS software v21.

## Results

The response rate was 78.25% (334 respondents); among these, 61.4% of respondents were females, the remaining 38.6% were males, and the mean age was 56.39 years (*SD* = 10.21 years). The participants’ socio-demographic analysis showed 91.9% non-smokers, 67% married, and 54.2% unemployed. The average total diabetes distress score was 2.43 ± 1.01, with the emotional burden domain being the highest at 2.63 ± 1.29 (Table 1).

**TABLE 1:** Summary of descriptive statistics of socio-demographics, clinical factors, diabetes distress, and its domains.

Predictors	*N*	Mean (*SD*)
Age	327	56.39 years (10.21)
Height	225	154.46 cm (30.61)
Weight	239	80.76 kg (19.10)
BMI	218	31.98kg/m^2^ (8.54)
HBa1c level	229	7.98% (2.51)
Cholesterol level	224	4.16 mmol/L (1.40)
LDL level	221	2.36 mmol/L (1.32)
HDL level	223	1.74 mmol/L (9.31)
Triglyceride level	230	3.02 mmol/L (14.20)
Systolic blood pressure	243	132.52 mmHg (18.00)
Diastolic blood pressure	243	73.16 mmHg (11.79)
SDSCA total	335	3.35 (1.42)
Total DDS	334	2.43(1.01)
Emotional Burden	334	2.63(1.29)
Physician related distress	334	2.31(1.44)
Regimen related distress	334	2.48(1.16)
Interpersonal distress	334	2.23(1.24)

*HDL, high-density lipoprotein; LDL, low-density lipoprotein; HBa1c, hemoglobin A1c; BMI, Body Mass Index; SDSCA, Summary of Diabetes Self- Care Activities.*

The mean scores for the SADDS-17 subdomains, including emotional burden, physician-related distress, regimen-related distress, and interpersonal distress, were 2.63 ± 1.29, 2.31 ± 1.44, 2.48 ± 1.16, and 2.23 ± 1.24, respectively (Table 1). The average body-mass index (BMI) of participants was 31.98 (8.54 kg/m^2^), and the majority (63.3%) were on oral hypoglycemics treatment.

**FIGURE 1:**
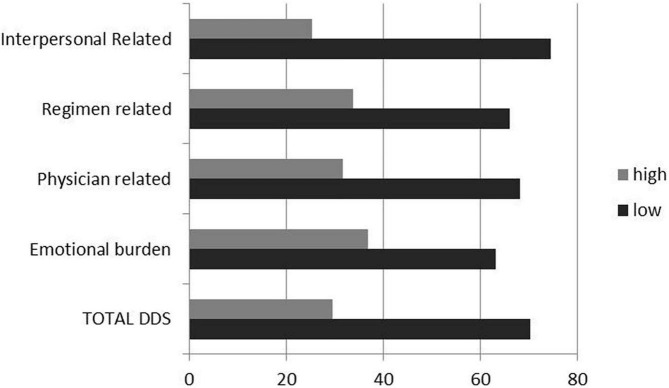
Distribution of respondents according to the level of diabetes distress (*N* = 334).

The respondents’ prevalence of diabetes distress was 29.4%, as calculated from total SADDS-17 scores. The distribution of participants across the four subdomains is shown in Figure 1. The prevalence of anxiety and depression were 12.4 and 23.5%, respectively, as estimated from the HADS scores (Table 2). Based on the WHOQOL-BREF transformed scores, the quality of life was recorded as 62.7%.

**TABLE 2:** Prevalence of depression, anxiety, and diabetes distress.

Factor	% (*N*)
Depression	23.5(319)
Anxiety	12.4(322)
Diabetes distress	29.4(334)

The respondents’ mean prevalence of diabetes distress among different groups revealed 2.81 ± 1.14 for the 15,000–20,000 SAR average monthly family income group, 2.64 ± 0.96 for the singles group in social status, 2.57 ± 0.98 for the businessman group in occupation, 2.52 ± 1.03 for the 5–9 family members under care group, 2.51 ± 1.12 for the bachelor’s degree group in educational level, 2.47 ± 1.04 for the 41–60 years age group, and 2.47 ± 1.04 for the female group in gender (Table 3).

**TABLE 3:** Summary of univariate analysis between diabetes distress scores and socio-demographic characteristics.

Predictors	Cutoffs	Frequency (%)	Total DDS	Emotional burden	Physician related	Regimen related	Interpersonal Related	Level of diabetes distress n (%)		*p*
								Little/No distress (<3)	High distress (3+)	
Age	21–40	21(6.4)	2.37(1.03)	2.59(1.36)	1.82(1.06)	2.86(1.5)	2.1(1.23)	14(66.67)	7(33.33)	ns
	41–60	206(63.0)	2.47(1.04)	2.69(1.34)	2.4(1.55)	2.47(1.15)	2.29(1.26)	139(68.14)	65(31.86)	
	61–80	97(27.7)	2.34(0.95)	2.45(1.12)	2.22(1.26)	2.4(1.09)	2.13(1.26)	75(78.13)	21(21.88)	
	81+	3(0.9)	2.42(1.01)	2.61(1.28)	2.31(1.45)	2.48(1.16)	2.22(1.25)	2(66.67)	1(33.33)	
	Total	324(100)	2.42(1.01)	2.61(1.28)	2.31(1.45)	2.48(1.16)	2.22(1.25)	230(70.99)	94(29.01)	
Gender	Male	129(38.6)	2.34(1.01)	2.36(1.13)	2.25(1.37)	2.52(1.18)	2.06(1.15)	93(73.23)	34(26.77)	(*) *P* < 0.05
	Female	205(61.4)	2.47(1)	2.78(1.36)	2.34(1.48)	2.44(1.14)	2.32(1.28)	141(69.12)	63(30.88)	
	Total	331(100)	2.42(1.01)	2.62(1.29)*	2.31(1.44)	2.47(1.15)	2.22(1.24)	234(70.69)	97(29.31)	
Social status	Single	33(9.8)	2.64(0.96)	2.69(1.24)	2.4(1.25)	2.87(1.19)	2.51(1.1)	19(59.38)	13(40.63)	ns
	Married	225(67)	2.37(1.04)	2.52(1.25)	2.29(1.49)	2.47(1.21)	2.12(1.21)	162(72.65)	61(27.35)	
	Widow	55(16.4)	2.58(0.98)	3.01(1.41)	2.33(1.4)	2.44(1)	2.42(1.35)	36(65.45)	19(34.55)	
	Divorced	23(6.8)	2.42(0.8)	2.71(1.37)	2.36(1.35)	2.25(0.86)	2.45(1.46)	17(73.91)	6(26.09)	
	Total	333(100)	2.43(1.01)	2.63(1.29)	2.32(1.44)	2.48(1.16)	2.23(1.24)	234(70.27)	99(29.73)	
Education level	Illiterate	92(27.7)	2.49(1.03)	2.99(1.43)	2.25(1.4)	2.36(1.06)	2.37(1.4)	62(68.13)	29(31.87)	(*) *P* < 0.05
	Elementary	44(13.3)	2.3(0.9)	2.63(1.34)	2.13(1.41)	2.27(1.06)	2.08(1.08)	31(72.09)	12(27.91)	
	Secondary	57(17.2)	2.31(0.95)	2.48(1.31)	2.15(1.43)	2.47(1.2)	2.06(1.21)	46(80.7)	11(19.3)	
	High school	85(25.6)	2.49(1)	2.5(1.18)	2.5(1.4)	2.57(1.19)	2.35(1.18)	57(67.06)	28(32.94)	
	Bachelor	44(13.3)	2.51(1.12)	2.49(1.09)	2.51(1.63)	2.66(1.2)	2.19(1.23)	29(67.44)	14(32.56)	
	Post graduate	10(3)	2.42(1)	2.63(1.3)	2.3(1.43)	2.47(1.14)	2.23(1.24)	8(80)	2(20)	
	Total	329	2.42(1)	2.63(1.3)*	2.3(1.43)	2.47(1.14)	2.23(1.24)	233(70.82)	96(29.18)	
Occupation	Unemployed	182(54.2)	2.45(1)	2.77(1.35)	2.32(1.44)	2.42(1.11)	2.3(1.25)	127(70.17)	54(29.83)	ns
	Governmental sector employee	60(17.9)	2.45(1.04)	2.44(1.08)	2.38(1.49)	2.7(1.21)	2.06(1.1)	40(66.67)	20(33.33)	
	Business professional	21(6.3)	2.57(0.98)	2.59(1.2)	2.35(1.39)	2.58(1.01)	2.73(1.5)	12(60)	8(40)	
	Retired	66(19.6)	2.32(1.05)	2.47(1.34)	2.29(1.48)	2.33(1.21)	2.12(1.26)	49(75.38)	16(24.62)	
	Private sector employee	7(2.1)	2.34(0.65)	2.17(1.1)	1.79(0.78)	3.31(1.49)	1.67(0.96)	6(85.71)	1(14.29)	
	Total	333(100)	2.43(1.01)	2.63(1.29)	2.32(1.44)	2.48(1.16)	2.23(1.24)	234(70.27)	99(29.73)	
Family income	Less than 5,000 SAR	116 (37.1)	2.46(0.96)	2.85(1.39)	2.21(1.39)	2.4(1.12)	2.33(1.28)	79(68.7)	36(31.3)	ns
	From 5,000 to 10,000 SAR	85(27.2)	2.38(0.95)	2.59(1.18)	2.3(1.45)	2.45(1.18)	2.15(1.21)	61(72.62)	23(27.38)	
	From 10,000 to 15,000 SAR	62(19.8)	2.35(0.98)	2.45(1.1)	2.35(1.5)	2.5(1.1)	2.01(1.03)	47(75.81)	15(24.19)	
	From 15,000 to 20,000 SAR	35(11.2)	2.81(1.14)	2.76(1.26)	2.78(1.55)	2.93(1.24)	2.54(1.42)	18(52.94)	16(47.06)	
	More than 20,000 SAR	15(4.8)	2.34(0.95)	2.2(1.04)	2.37(1.56)	2.59(1)	2.02(1.17)	11(73.33)	4(26.67)	
	Total	310(100)	2.45(0.99)	2.66(1.26)	2.33(1.45)	2.5(1.14)	2.23(1.23)	216(69.68)	94(30.32)	
Number of family members under your care	<5	147(45.2)	2.36(0.96)	2.57(1.2)	2.17(1.39)	2.46(1.11)	2.12(1.19)	110(74.83)	37(25.17)	ns
	5–9	150(46.2)	2.52(1.03)	2.73(1.33)	2.51(1.46)	2.5(1.17)	2.31(1.26)	94(63.95)	53(36.05)	
	10–14	23(7.1)	2.15(0.89)	2.38(1.38)	1.84(1.25)	2.1(0.88)	2.25(1.44)	19(82.61)	4(17.39)	
	15 – 19	3(0.9)	1.51(0.07)	1.6(0.6)	1.58(1.01)	1.33(0.42)	2(0.67)	3(100)	0(0)	
	20+	2(0.6)	2.26(0.12)	2.7(0.14)	2.25(1.77)	2.6(1.13)	2.33(0.47)	2(100)	0(0)	
	Total	322(100)	2.41(0.99)	2.62(1.27)	2.3(1.43)	2.44(1.13)	2.22(1.23)	228(70.81)	94(29.19)	

In addition, the highest percentages of diabetes distress symptoms were recorded among the no diabetes medication group (50%), family income group of 15,000–20,000 SAR (47.06%), the singles group (40.63%) in social status, and business professional group (40%) in occupation (Tables 3, 4).

The univariate analysis results between diabetes distress scores and socio-demographic characteristics are shown in Table 3. Education level and gender were revealed to be significantly associated with emotional burden related to diabetes distress. However, no association was revealed between the other variables (age, social status, occupation, family income, number of family members under your care) and any of the DDS subdomains (Table 3).

In the clinical variables, higher diabetes distress levels were observed in the no diabetes medication group (2.98 ± 1.42), underweight (<18.5) in BMI group (2.94 ± 0.86), the smokers’ group (2.63 ± 1.05), and the 30–39 years duration of DM group (2.62 ± 0.98) (Table 4). Table 4 shows a univariate analysis between distress scores and clinical characteristics. The medication of DM, VAS scores, and PHQ 15 scores exhibited a significant association with total DDS and emotional burden related to diabetes distress. Thyroid conditions showed a significant association with emotional burden related to diabetes distress. HBA1c level and PHQ 15 scores revealed a significant association with physician-related diabetes distress. VAS scores, thyroid conditions, and PHQ 15 scores showed a significant association with regimen-related diabetes distress. PHQ 15 scores showed an association with interpersonal-related diabetes distress. However, there was no association between other clinical variables (BMI, smoking, duration of diabetes, retinopathy, neuropathy, PAD, macular, edema, thyroid conditions, stroke, MI, other heart conditions, nervous system), and any of the DDS sub-domains.

**TABLE 4:** Summary of univariate analysis between Distress scores and clinical characteristics.

Predictors	Cutoffs	Frequency (%)	Total DDS	Emotional burden	Physician related	Regimen related	Interpersonal related	Level of diabetes distress *n* (%)		*p*
								Little/No distress (<3)	High distress (3+)	
HBa1c level	<7.0	61(26.6)	2.62(1.11)	2.82(1.46)	2.66(1.59)	2.58(1.24)	2.42(1.36)	37(60.66)	24(39.34)	(*) *P* < 0.05
	7+	166(73.4)	2.36(0.99)	2.52(1.22)	2.22(1.41)	2.45(1.14)	2.12(1.23)	119(71.69)	47(28.31)	
	Total	227(100)	2.43(1.03)	2.6(1.29)	2.34(1.47)*	2.49(1.17)	2.2(1.27)	156(68.72)	71(31.28)	
Smoking	Non-Smoker	308(91.9)	2.4(1)	2.59(1.28)	2.3(1.43)	2.44(1.14)	2.21(1.24)	217(70.92)	89(29.08)	ns
	Smoker	27(8.1)	2.63(1.05)	2.97(1.33)	2.37(1.58)	2.79(1.25)	2.33(1.13)	18(69.23)	8(30.77)	
	Total	332(100)	2.42(1)	2.62(1.29)	2.3(1.44)	2.47(1.15)	2.22(1.23)	235(70.78)	97(29.22)	
Duration of diabetes (years)	<10.0	139(42.6)	2.47(1)	2.63(1.34)	2.38(1.43)	2.53(1.17)	2.24(1.24)	89(64.96)	48(35.04)	ns
	10.0–19.9	109(33.4)	2.34(0.97)	2.51(1.25)	2.11(1.29)	2.44(1.15)	2.28(1.26)	85(78.7)	23(21.3)	
	20.0–29.9	57(17.5)	2.44(1.04)	2.71(1.23)	2.45(1.67)	2.42(1.08)	2.16(1.22)	39(68.42)	18(31.58)	
	30.0 – 39.9	16(4.9)	2.62(0.98)	2.98(1.26)	2.66(1.53)	2.41(1.34)	2.27(1.16)	9(56.25)	7(43.75)	
	40.0+	5(1.5)	2.42(0.89)	2.72(0.72)	1.9(1.23)	2.84(1.24)	1.93(1.23)	4(80)	1(20)	
	Total	323(100)	2.43(0.99)	2.62(1.28)	2.31(1.43)	2.48(1.15)	2.24(1.24)	226(69.97)	97(30.03)	
Medication for DM	No diabetic medication	10(4.1)	2.98(1.42)	3.6(1.63)	2.65(1.86)	2.64(1.65)	2.9(1.94)	5(50)	5(50)	(*) *P* < 0.05
	Oral hypoglycemic only	155(63.3)	2.29(0.94)	2.43(1.23)	2.23(1.37)	2.4(1.13)	2.06(1.1)	115(75.16)	38(24.84)	
	Insulin only	23(9.4)	2.81(1)	3.02(1.36)	2.87(1.67)	2.85(1.08)	2.48(1.35)	12(52.17)	11(47.83)	
	Both oral and insulin	57(23.3)	2.46(1.05)	2.71(1.2)	2.2(1.45)	2.47(1.15)	2.31(1.39)	38(66.67)	19(33.33)	
	Total	243(100)	2.41(1.01)*	2.6(1.27)*	2.3(1.45)	2.47(1.15)	2.19(1.25)	170(69.96)	73(30.04)	
VAS	<8	134(40.9)	2.59(0.89)	2.79(1.22)	2.41(1.32)	2.74(1.1)	2.32(1.15)	91(67.91)	43(32.09)	(*) *P* < 0.001
	8+	194(59.1)	2.28(1.03)	2.48(1.3)	2.2(1.49)	2.27(1.13)	2.12(1.25)	140(72.92)	52(27.08)	
	Total	326(100)	2.41(0.98)*	2.61(1.27)*	2.29(1.43)	2.47(1.14)*	2.2(1.21)	231(70.86)	95(29.14)	
BMI	<18.50	4(1.8)	2.94(0.86)	2.85(1.53)	3.69(1.68)	2.7(0.62)	2.5(1.55)	2(50)	2(50)	ns
	18.50–24.99	22(10.1)	2.57(1.13)	2.6(1.14)	2.5(1.61)	2.82(1.29)	2.33(1.29)	14(66.67)	7(33.33)	
	25.00–29.99	74(33.9)	2.36(0.98)	2.4(1.2)	2.35(1.39)	2.5(1.2)	2.06(1.06)	54(73.97)	19(26.03)	
	30.00+	118(54.1)	2.41(1.01)	2.66(1.3)	2.22(1.45)	2.44(1.11)	2.28(1.37)	82(69.49)	36(30.51)	
	Total	218(100)	2.42(1.01)	2.57(1.26)	2.32(1.46)	2.5(1.15)	2.21(1.26)	152(70.37)	64(29.63)	
PHQ 15	<5.00	75(22.3)	1.98(0.93)	2.16(1.25)	1.86(1.21)	2.08(1.13)	1.79(0.99)	63(87.5)	9(12.5)	(*) *P* < 0.001
	5.00–14.00	162(48.1)	2.43(0.96)	2.51(1.2)	2.34(1.45)	2.57(1.08)	2.2(1.16)	113(69.75)	49(30.25)	
	15.00–29.00	100(29.7)	2.74(1.03)	3.15(1.3)	2.59(1.51)	2.63(1.24)	2.59(1.42)	59(59)	41(41)	
	Total	334(100)	2.43(1.01)*	2.63(1.29)*	2.31(1.44)*	2.48(1.16)*	2.23(1.24)*	235(70.36)	99(29.64)	
Retinopathy	No	91(27)	2.42(1)	2.63(1.26)	2.31(1.43)	2.45(1.13)	2.21(1.2)	62(68.89)	28(31.11)	ns
	Yes	30(8.9)	2.3(0.95)	2.41(1.13)	2.08(1.34)	2.51(1.13)	2.03(1.24)	22(75.86)	7(24.14)	
	Not known	215(63.8)	2.45(1.02)	2.66(1.33)	2.35(1.46)	2.49(1.17)	2.26(1.27)	151(70.23)	64(29.77)	
	Total	334(100)	2.43(1.01)	2.63(1.29)	2.31(1.44)	2.48(1.16)	2.23(1.24)	235(70.36)	99(29.64)	
Neuropathy	No	176(52.2)	2.44(1.06)	2.55(1.26)	2.36(1.45)	2.56(1.21)	2.23(1.27)	120(68.57)	55(31.43)	ns
	Yes	24(7.1)	2.58(0.72)	3.15(1.15)	2.33(1.47)	2.67(0.88)	2.07(0.86)	16(69.57)	7(30.43)	
	Not known	137(40.7)	2.39(0.99)	2.64(1.34)	2.25(1.43)	2.35(1.12)	2.25(1.26)	99(72.79)	37(27.21)	
	Total	334(100)	2.43(1.01)	2.63(1.29)	2.31(1.44)	2.48(1.16)	2.23(1.24)	235(70.36)	99(29.64)	
PAD	No	187(55.5)	2.4(1.03)	2.58(1.3)	2.28(1.41)	2.46(1.17)	2.19(1.25)	131(70.43)	55(29.57)	ns
	Yes	14(4.2)	2.87(0.82)	2.87(0.89)	2.64(1.54)	3.24(1.01)	2.38(1.2)	8(57.14)	6(42.86)	
	Not known	135(40.1)	2.42(0.99)	2.66(1.33)	2.29(1.44)	2.43(1.14)	2.27(1.24)	96(72.18)	37(27.82)	
	Total	334(100)	2.43(1.01)	2.63(1.29)	2.31(1.44)	2.48(1.16)	2.23(1.24)	235(70.57)	98(29.43)	
Macular edema	No	107(31.8)	2.41(1)	2.63(1.25)	2.26(1.39)	2.44(1.13)	2.26(1.22)	73(68.87)	33(31.13)	ns
	Yes	4(1.5)	2.85(1.56)	2.55(1.67)	2.75(1.62)	3.05(1.48)	2.67(1.89)	2(50)	2(50)	
	Not known	224(66.5)	2.42(1)	2.61(1.29)	2.32(1.46)	2.49(1.17)	2.21(1.25)	160(71.75)	63(28.25)	
	Total	3334(100)	2.43(1.01)	2.63(1.29)	2.31(1.44)	2.48(1.16)	2.23(1.24)	235(70.57)	98(29.43)	
**Past medical conditions**										
Thyroid conditions	No	191(78.3)	2.48(1.01)	2.67(1.28)	2.34(1.47)	2.59(1.18)	2.23(1.26)	128(67.72)	61(32.28)	(*) *P* < 0.05
	Yes	53(21.7)	2.18(0.96)	2.39(1.24)	2.17(1.34)	2.07(0.94)	2.07(1.22)	41(77.36)	12(22.64)	
	Total	244(100)	2.41(1.01)*	2.6(1.27)	2.3(1.45)	2.48(1.15)*	2.19(1.25)	169(69.83)	73(30.17)	
Stroke	No	208(61.7)	2.46(1.04)	2.64(1.31)	2.39(1.48)	2.5(1.17)	2.24(1.28)	138(66.99)	68(33.01)	ns
	Yes	16(4.7)	2.23(0.71)	2.6(1.01)	1.69(0.97)	2.68(1.06)	1.88(0.57)	14(87.5)	2(12.5)	
	Not known	113(33.5)	2.4(0.98)	2.61(1.3)	2.26(1.4)	2.42(1.15)	2.26(1.24)	83(74.11)	29(25.89)	
	Total	334(100)	2.43(1.01)	2.63(1.29)	2.31(1.44)	2.48(1.16)	2.23(1.24)	235(70.36)	99(29.64)	
MI	No	195(57.9)	2.41(1.03)	2.58(1.31)	2.31(1.44)	2.47(1.14)	2.21(1.28)	134(69.07)	60(30.93)	ns
	Yes	29(8.6)	2.6(0.95)	2.94(1.09)	2.34(1.45)	2.77(1.26)	2.15(1.02)	19(67.86)	9(32.14)	
	Not known	113(33.5)	2.43(0.99)	2.63(1.31)	2.31(1.44)	2.43(1.15)	2.28(1.23)	82(73.21)	30(26.79)	
	Total	334(100)	2.43(1.01)	2.63(1.29)	2.31(1.44)	2.48(1.16)	2.23(1.24)	235(70.36)	99(29.64)	
Other heart conditions	No	112(33.2)	2.39(1.07)	2.48(1.3)	2.36(1.5)	2.49(1.17)	2.21(1.25)	75(66.96)	37(33.04)	ns
	Yes	121(35.8)	2.46(0.97)	2.77(1.25)	2.27(1.42)	2.5(1.16)	2.18(1.25)	84(70.59)	35(29.41)	
	Not known	104(30.9)	2.43(0.98)	2.62(1.32)	2.3(1.4)	2.45(1.15)	2.31(1.24)	76(73.79)	27(26.21)	
	Total	334(100)	2.43(1.01)	2.63(1.29)	2.31(1.44)	2.48(1.16)	2.23(1.24)	235(70.36)	99(29.64)	
Nervous system	No	236(96.7)	2.42(1.02)	2.62(1.28)	2.32(1.44)	2.49(1.16)	2.2(1.24)	163(69.66)	71(30.34)	ns
	Yes	8(3.3)	2.11(0.64)	2.25(1.1)	1.97(1.72)	2.08(0.69)	2.04(1.64)	6(75)	2(25)	
	Total	244(100)	2.41(1.01)	2.6(1.27)	2.3(1.45)	2.48(1.15)	2.19(1.25)	169(69.83)	73(30.17)	

*PAD, peripheral arterial disease; MI, myocardial infarction; DM, diabetes mellitus; ns, not significant.*

A statistically significant positive correlation between the HADS scale score (*r* = 0.287, *p* < 0.01) and the total SADDS-17 scores (*r* = 0.433, *p* < 0.01) related to the depression and anxiety and significant positive correlation with the other four sub-domains of SADDS-17 (Table 5).

**TABLE 5:** Correlation of SADDS-17 scores with other participant demographics and clinical variables.

	Total DDS	Emotional burden	Physician related	Regimen related	Interpersonal related
SBP	−0.144*	–0.118	−0.141*	–0.117	–0.106
DBP	–0.120	–0.096	0.003	−0.149*	−0.133*
Height	–0.037	−0.160*	–0.028	0.136*	–0.092
Weight	–0.040	0.015	−0.140*	–0.035	0.024
HADS depression	0.287**	0.355**	0.158**	0.221**	0.167**
HADS anxiety	0.433**	0.487**	0.300**	0.241**	0.359**
SDSCA total	–0.057	0.025	–0.028	−0.183**	0.056
WHOQOL Total	−0.229**	−0.214**	−0.143**	−0.168**	−0.230**

* Significant at p < 0.05. **Significant at p < 0.01

A statistically significant negative correlation was seen between the systolic blood pressure (SBP) with the total SADDS-17 (*r* = −0.144, *p* < 0.05) and physician-related distress (*r* = −0.141, *p* < 0.05). Similarly, there was a significant negative correlation between diastolic blood pressure (DBP) with regimen-related distress (*r* = −0.149, *p* < 0.05) and interpersonal-related distress (*r* = −0.149, *p* < 0.05). A significant negative correlation was revealed between height and emotional burden distress (*r* = −0.16, *p* < 0.05) and between weight and physician-related distress (*r* = −0.14, *p* < 0.05).

The PHQ15 scores showed significant positive correlations with the total SADDS-17 scores (*r* = 0.288, *p* < 0.01) and each of the four subdomains. On the contrary, WHOQOL scores revealed significant negative correlations (*r* = −0.229, *p* < 0.01) with the SADDS-17 scores (Table 5). The SDSCA scores showed a significant negative correlation with regimen-related distress (*r* = −0.183, *p* < 0.01).

Among all variables associated with the total diabetes distress scores, PHQ 15 scores, SBP, HADS anxiety and depression scores, and WHOQOL total scores were shown to be independent variables. In the variables associated with the emotional burden, PHQ 15 scores, SBP, HADS anxiety and depression scores, and WHOQOL total scores were revealed as independent variables. For variables associated with physician-related diabetes distress, weight, and HADS anxiety scores were shown to be independent variables. Similarly, in the variables associated with regimen-related diabetes distress, VAS scores, PHQ 15 scores, thyroid conditions, HADS anxiety and depression scores, SDSCA total scores, and WHOQOL total scores were identified as the independent variables. Finally, the variables associated with interpersonal-related diabetes distress, PHQ 15 scores, HADS anxiety and depression scores, and WHOQOL total scores were revealed as independent variables (Table 6).

**TABLE 6:** Multivariate analysis of variables associated with diabetes distress.

	Variables	*P*-value
Total DDS	Visual analog scale	0.062
	Diabetes mellitus medication	0.584
	PHQ 15	0.000*
	Thyroid conditions	0.056
	Systolic blood pressure	0.025*
	HADS anxiety	0.000*
	HADS depression	0.000*
	WHOQOL total	0.002*
Emotional burden-related distress	Gender	0.128
	Educational level	0.028*
	Diabetes mellitus medication	0.801
	Visual analog scale	0.100
	PHQ 15	0.000*
	Height	0.014*
	HADS anxiety	0.000*
	HADS depression	0.000*
	WHOQOL total	0.020*
Physician-related	HBa1c level	0.076
	PHQ 15	0.075
	Systolic blood pressure	0.112
	Weight	0.022*
	HADS anxiety	0.000*
	HADS depression	0.067
	WHOQOL total	0.055
Regimen-related	Visual analog scale	0.000*
	PHQ 15	0.014*
	Thyroid conditions	0.007*
	Diastolic blood pressure	0.063
	HADS anxiety	0.000*
	HADS depression	0.000*
	SODSCA total	0.018*
	WHOQOL total	0.029*
Interpersonal-related	PHQ 15	0.000*
	Diastolic blood pressure	0.081
	HADS anxiety	0.000*
	HADS depression	0.022*
	WHOQOL total	0.001*

* Significant at p < 0.05.

## Discussion

Diabetes distress is a common health problem worldwide. The present findings demonstrated that 29.4% of people with diabetes had high distress, consistent with a recent systematic review and meta-analysis, where females were a higher proportion (Perrin et al., 2017). However, the prevalence of diabetes distress was relatively a little higher than in other local studies in Taif (25%) and Jazan (22.3%) cities (Aljuaid et al., 2018; Alzughbi et al., 2020). Consistent with an earlier study, the emotional burden had the highest impact on measuring diabetes distress (2.63 ± 1.29) among the four SADDS-17 subdomains (Onyenekwe et al., 2020).

In line with a previous study (Forsander et al., 2017), gender is the major factor influencing the levels of diabetes distress (emotional burden). Our findings indicate that females appeared particularly vulnerable, which may need more attention and support against diabetes distress. In parallel, a meta-analysis showed that females had higher diabetic distress than males and indicated that gender is the major socio-demographic factor associated with diabetes distress levels (Perrin et al., 2017).

The status of occupation plays a significant role in the development of depression and anxiety. People who are unemployed are more likely to experience anxiety and depression. The relationship between unemployment, depression, and anxiety can be described in two ways: sociological and economic. Individuals who are unemployed lack sociological functions such as time structure, status and identity, social contacts, participation in common goals, and consistent activity (Palizgir et al., 2013; Nordenmark and Strandh, 2016). In our study, over half of the individuals (54.2%) were unemployed, although this finding was unexpectedly unrelated to diabetes distress. Our findings contradict prior research, which revealed that unemployed people had higher levels of diabetes-related distress than employed people, ranging from 70 to 83.1% (Palizgir et al., 2013; Ramkisson et al., 2016; Dogra et al., 2017).

Our findings revealed that the illiterate group registered the highest emotional burden distress at 2.99 ± 1.43. Many studies suggested an inversely proportional relationship between the patients’ educational levels and diabetes distress levels. Those with low educational levels are more likely to experience more distress (van Bastelaar et al., 2010; Papelbaum et al., 2011; Graue et al., 2012; Baradaran et al., 2013). Similarly, another report indicated that the odds of becoming distressed over time were higher at a lower educational level (Fisher et al., 2009). Studies have also reported that low education leads to poor knowledge about the illness and its complications, which increases the risk of poor dietary habits, poor compliance to medication, and fewer health check-ups (Gahlan et al., 2018). Interestingly, consistent with the above findings, in our study, participants’ educational level revealed a notable influence on the level of diabetes distress (emotional burden). On the contrary, Wardian and Sun have reported that education was not significantly related to diabetes distress due to the lack of variation in education levels (Wardian and Sun, 2014). However, diabetes education requires individuals to increase self-efficacy and management of diabetes distress.

Consistent with previous findings (Fisher et al., 2008; van Bastelaar et al., 2010), HBA1c levels and diabetes distress had a significant negative correlation, and patients with higher (>7) HBA1c were experiencing lesser distress. On the contrary, a positive correlation between depression and HbA1c levels among diabetes patients was reported (Papelbaum et al., 2011), which may require further detailed investigation.

In line with prior international reports (Aikens, 2012), the influence of DM medication regimens on the level of diabetes distress was statistically significant at (*p* < 0.05). Previous quantitative research suggested that insulin-treated T2DM patients have experienced significantly higher diabetes distress, particularly regimen-related distress (Baek et al., 2014). In parallel, patients treated with insulin in our study had the highest amount of regimen-related distress at 2.85 ± 1.08. Surprisingly, the study indicated that insulin distress could take multiple forms, such as acute, sub-acute, or chronic insulin distress (Kalra and Balhara, 2018). However, in our present study, patients without any DM medication had the highest total diabetes distress (2.98 ± 1.42) than other medication groups.

Martino et al. (2019) looked at the impact of anxious and depressive symptoms, time since T2DM diagnosis, and metabolic control on health-related quality of life (HRQoL), focusing on physical and mental component summaries. In a chronic condition like T2DM, both anxiety levels and time since diagnosis play a predictive role in HRQoL. Higher Physical Component Summary (PCS) values, which indicate a superior HRQoL in the physical component, were linked to lower anxiety levels and a shorter period since T2DM diagnosis. Furthermore, greater Mental Component Summary (MCS) values, which indicate a superior HRQoL in the mental component, were linked to a reduction in anxiety and depression symptoms, but not to diabetes duration or metabolic control (Martino et al., 2019). Moreover, Bai et al. (2018) reported that both anxiety and depression were strongly correlated with diabetes distress. Anxiety has been attributed to fear of insulin injection and is common among people with diabetes (Abu Hassan et al., 2013). In this context, an extreme level of fear of self-injection is associated with high levels of diabetes distress, low general well-being, and psychological comorbidity, as well as poor adherence to the diabetes treatment regimen (Mollema et al., 2001; Abu Hassan et al., 2013). In agreement with the above findings, our finding showed a strong positive correlation between anxiety scores and the level of diabetes distress (*r* = 0.433, *p* < 0.01).

The total diabetes distress scores and sub-scales revealed a significant positive correlation with participants’ depressive scores as calculated from the HADS scale. The total DDS score showed (*r* = 0.287, *p* < 0.01) that depression was an independent variable to the total SADDS-17. Furthermore, Baradaran et al. (2013) reported a high percentage of diabetes distress patients diagnosed with depression. Adequate studies indicated a significant correlation between diabetes distress and depression symptoms among DM patients (Fisher et al., 2010; Chew et al., 2016).

Among past medical conditions, thyroid conditions had a statistically significant (*p* < 0.05) influence on the level of diabetes distress, which attribute to the close link between thyroid conditions and depressive symptoms. In agreement with our study, the clinical report indicated that patients with thyroid disorders are more prone to develop depression than those without thyroid disorders (Hage and Azar, 2012).

An independent strong negative correlation was revealed between WHOQOL-BREF scores related to the quality of life and the total diabetes distress scores (*r* = −0.229, *p* < 0.01). Inconsistent with our study, Morisky et al. (2008) reported that lower health-related quality of life was strongly associated with a higher level of emotional distress, which indicated that quality of life important predictor of diabetes distress.

The PHQ15 scores showed a significant positive correlation with the total SADDS-17 (*r* = 0.288, *p* < 0.01) and all four subdomains. Furthermore, we found an independent variable for the total SADDS-17 and its subdomains except (physician-related distress). Most interestingly, to the best of our knowledge, the relation between somatic symptoms and diabetes distress has not been addressed yet. However, it could be attributed to the link between somatic symptoms and quality of life. Alosaimi et al. indicated that the severity of somatic symptoms is correlated with a low quality of life (Alosaimi et al., 2019). Zhu et al. (2012) reported that an independent association exists between PHQ15 scores and depressive symptoms.

The SDSCA scores revealed a significant negative correlation with the regimen-related level of diabetes distress (*r* = −0.183, *p* < 0.05). Khan and Choudhary (2018) reported that high levels of diabetes distress might be attributed to reduced self-care management (Devarajooh and Chinna, 2017). We believe that fewer worries could be achieved if patients with diabetes are more adherent to self-care (exercise, dietary habits, blood glucose monitoring, and foot care); hence, less regimen-related diabetes distress could be experienced.

## Limitations and Recommendations

Because the present study was a cross-sectional study, it has the following limitations that are mainly related to the study design. First, the temporal relationship between diabetes distress and certain factors could not confirm causality. Second, because this study used a convenience sample method from a single institute, the results cannot be applied to the entire Saudi Arabian population. Response bias may have occurred as a result of the sampling procedure. In order to resolve these limitations, further studies on a wider national scale are required to estimate the magnitude of diabetes distress in Saudi, as well as the use of more diversified approaches.

## Conclusion

Our present study revealed that diabetes distress prevalence is alarmingly high among patients in Saudi Arabia, Riyadh. Our findings provide evidence that physical symptoms, quality of life, depression, and anxiety are notable predictors of diabetes distress.

## Data Availability Statement

The raw data supporting the conclusions of this article will be made available by the authors, without undue reservation.

## Ethics Statement

The studies involving human participants were reviewed and approved by the Research Ethics Committee at King Saud University. The participants provided their written informed consent to participate in the study.

## Author Contributions

MAB and FDA designed the study. AFA, AAA, OAA, MKA, KSA, AMA, and MTA facilitated data gathering. MAB, FDA, AFA, AAA, OAA, MKM, KSA, AMA, and MTA performed the data analysis and drafted the manuscript. All authors contributed to the interpretation of results and the revision of the manuscript, as well as approving the final manuscript.

## Conflict of Interest

The authors declare that the research was conducted in the absence of any commercial or financial relationships that could be construed as a potential conflict of interest.

## Publisher’s Note

All claims expressed in this article are solely those of the authors and do not necessarily represent those of their affiliated organizations, or those of the publisher, the editors and the reviewers. Any product that may be evaluated in this article, or claim that may be made by its manufacturer, is not guaranteed or endorsed by the publisher.
